# Dissemination of the high-risk cloneST147 carbapenem-resistant *klebsiella pneumoniae* from a local tertiary care hospital in the Republic of Korea

**DOI:** 10.1186/s12941-023-00601-2

**Published:** 2023-08-24

**Authors:** Jungsun Park, Eunkyung Shin, Gwang Rip Hwang, Min-Kyeong Kim, Seongjae Joo, Hyun Ju Jeong, Jin Seok Kim, Jaeil Yoo, Junyoung Kim

**Affiliations:** 1 Division of Bacterial Diseases, Bureau of Infectious Disease Diagnosis Control, Korea Disease Control and Prevention Agency, Cheongju-si, Chungcheongbuk-do Republic of Korea; 2 Division of Infectious Diseases Research, Gyeongsangbuk-do Metropolitan Government Research Institute of Public Health and Environment, Gyeongsangbuk-do, Republic of Korea; 3 Infectious Diseases Team, Seoul Metropolitan Government Research Institute of Public Health and Environment, Gwacheon-si, Gyeonggi-do Republic of Korea

**Keywords:** *Klebsiella pneumoniae*, ST147, Carbapenemase, NDM-1

## Abstract

**Background:**

The emergence of carbapenem-resistant *Enterobacterales* (CRE) infections is rapidly increasing and represents a serious public threat. In 2020, a total of 16,883 carbapenemase-producing *Enterobacterales* strains were collected; among these isolates, 21 strains were repeatedly isolated in a local tertiary care hospital.

**Methods:**

Antimicrobial susceptibility testing was performed using the broth microdilution method. All 21 strains of CRKP were analyzed by PFGE after *Xba*I digestion. The 21 CRKP strains were sequenced on the Illumina Miseq and Oxford Nanopore GridION platforms.

**Results:**

These 21 CRKP isolates showed an identical antimicrobial resistance profile, including resistance to ampicillin, carbapenems, cephems, chloramphenicol, fluoroquinolone, macrolides and trimethoprim/sulfamethoxazole. Based on whole-genome analysis, these 21 CRKP isolates shared a common genetic structure (*ISAba125*-*IS630*-*bla*_NDM−1_-*ble*_MBL_) and harbored additional resistance determinants (*bla*_OXA−1_, *bla*_CTX−M−15_, *bla*_SHV−11_, *bla*_SHV−67_, *aac(6’)-Ib-cr*, *qnrS1*, *OqxA*, *OqxB*, *catB3*, *mph(A)*, *sul1*, and *dfrA12*) and mutations in the quinolone resistance-determining regions of *gyrA* (S83I) and *parC* (S80I). These isolates belonged to the ST147 and KL64 capsular types, which were carried on IncFIB replicon plasmids. The 21 CRKP strains collected from one hospital were divided into five PFGE patterns, and they were closely related with a minimum similarity value of 95.2%. These isolates were found to be highly related based on the presence of between 2 and 27 SNPs.

**Conclusions:**

These findings indicate that NDM-1-producing *K. pneumoniae* ST147 may have been introduced via a common source, implying nosocomial transmission; furthermore, continuous monitoring is necessary to prevent endemic transmission.

**Supplementary Information:**

The online version contains supplementary material available at 10.1186/s12941-023-00601-2.

## Background

The emergence of carbapenem-resistant *Enterobacterales* (CRE) infections is rapidly accelerating and represents a serious public threat. Recently, among CRE infectious agents, *Klebsiella pneumoniae* has become one of the most common causative pathogens of hospital- and community-acquired infections in the Republic of Korea [1]. Additionally, the predominant carbapenemase genes associated with *K. pneumoniae* infections are *K. pneumoniae* carbapenemase (KPC), followed by New Delhi metallo-β-lactamase (NDM), which was reported in a previous study [2]. Carbapenem-resistant *K. pneumoniae* has a significant influence on endemic transmission.

Since the first published records from Hungary and Spain in 2008 and 2009, respectively [3, 4]. Previous reports have indicated the dissemination of the *K. pneumoniae*, especially, specific sequence types or clones, including ST147, that are related with dissemination and success of carbapenem-resistant *K. pneumoniae*; these strains are classified as high-risk STs or clones [5, 6]. Also, several studies have reported the dissemination of high-risk clones of carbapenem-resistant *K. pneumoniae* [7–10]. *K. pneumoniae* ST147 is an emerging high-risk clone because of its successful acquisition of multidrug resistance and high transmissibility, both of which are an important vehicle for dissemination [5].

The Korea Disease Control and Prevention Agency (KDCA) has collected and tested all CRE isolates to monitor antimicrobial resistance profiles and the associated resistance genes by the Korean Antimicrobial Resistance Monitoring System. We confirmed that persistent infections caused by NDM-1-producing *K. pneumoniae* ST147 occurred in a local tertiary care hospital in the Republic of Korea. In this study, we elucidate the molecular and epidemiological characterization of NDM-1-producing *K. pneumoniae* ST147 isolates.

## Methods

### Bacterial isolates

In 2020, a total of 16,883 carbapenemase-producing *Enterobacterales* strains were collected from local public health laboratories, including tertiary care hospitals and long-term care hospitals. The bacterial species were confirmed using a VITEK 2 automated system (bioMérieux, Marcy l’Etoile, France) with a VITEK®2 GN ID card. All isolates were screened by PCR sequencing for the presence of carbapenemase genes (*bla*_NDM_, *bla*_KPC_, *bla*_IMP_, *bla*_VIM_, *bla*_OXA_ and *bla*_GES_), as described previously [11]. 9,234 (54.7%) of isolates were confirmed to be carbapenem-resistant *Enterobacterales*. The most prevalent CRE species detected were the *Klebsiella pneumoniae* (n = 6,254, 67.7%), followed by *Escherichia coli* (n = 1,591, 17.2%) and *Enterobacter cloacae* (n = 337, 3.6%). The following carbapenemase genes were identified: *bla*_KPC− 2_ (n = 5,511, 75.6%), *bla*_NDM− 1_ (n = 878, 12%), *bla*_NDM− 5_ (n = 285, 3.9%) and *bla*_OXA− 181_ (n = 167, 2.3%). Among these isolates, only 496 strains (5.4%) were identified as NDM-1-producing *K. pneumoniae* and these strains were sporadically isolated from separate regions in distinct hospitals. During this period, we found that only 21 NDM-producing *K. pneumoniae* were persistently isolated from a single hospital between June and December. In this process, we found that 21 carbapenem-resistant *K. pneumoniae* (CRKP) strains were persistently isolated from a local tertiary care hospital. We have selected the 21 isolates for analysis of their molecular epidemiology associations.

### Antimicrobial susceptibility testing and conjugation assay

Antimicrobial susceptibility testing was performed using the broth microdilution method with customized Sensititre KRCDC2F and KORN panels (TREK Diagnostic Systems, United Kingdom) in accordance with the guidelines established by the Clinical and Laboratory Standards Institute (CLSI) [12]. The antimicrobial agents tested were ampicillin, cephems, carbapenems, fluoroquinolone, gentamicin, streptomycin, tetracycline, trimethoprim/sulfamethoxazole and chloramphenicol. Conjugal transfer of carbapenem resistance genes were examined using azide-resistant *E. coli* J53 as the recipient strain. 21 *bla*_NDM−1_ positive CRKP isolates were selected for this assay. Donor and recipient cells from Luria-Bertani broth cultures were mixed in a ratio of 1:5 and transconjugants were selected on MacConkey agar plates (Difco, USA) supplemented with imipenem (1 mg/L) and sodium azide (200 mg/L). Carriage of *bla*_NDM–1_ in the transconjugant was confirmed by PCR and MICs.

### Pulsed-field gel electrophoresis (PFGE)

All 21 strains of CRKP were analyzed by pulsed-field gel electrophoresis (PFGE) after *Xba*I digestion according to the PulseNet International protocol (https://pulsenetinternational.org/). The genetic relatedness between PFGE patterns was calculated by using BioNumerics v7.6 (Applied Maths, Sint-Martens-Latem, Belgium).

### Whole-genome sequencing (WGS)

Genomic DNA of the 21 isolates was isolated using a Blood and Tissue Kit (Qiagen, Stockach, Germany) according to the manufacturer’s protocol. Short-read DNA libraries were prepared using an Illumina Nextera Flex library preparation kit and sequenced on a MiSeq sequencer (Illumina, San Diego, CA, USA). A long-read GridION sequencing library was prepared by using a ligation sequencing kit (SQK-LSK109), and sequencing was carried out using a version R9.4.1 flow cell (FLO-MIN 106D).

### WGS analysis

The contigs of genomic sequences were assembled with a minimum contig size threshold of 200 bp using the de novo assembly tool within the CLC genomic workbench 21.0.3. Assembled sequences were analyzed for resistance genes (ResFinder 4.1), sequence type (MLST 2.0) and plasmid replicon types (PlasmidFinder 2.1) using bioinformatics web tools available from the Center for Genomic Epidemiology (CGE) website (https://www.genomicepidemiology.org/). The capsular type and virulence genes of these isolates were confirmed using the Bacterial Isolate Genome Sequence Database (https://bigsdb.pasteur.fr/klebsiella/). Single-nucleotide polymorphisms (SNPs) of the 21 CRKP isolates were identified using CSI phylogeny 1.4 (https://cge.food.dtu.dk/services/CSIPhylogeny/) by comparison with the reference strain *K. pneumoniae* KP5 (GenBank accession no. CP012426) [13]. The phylogenetic analysis was performed by aligning the whole genome of *K. pneumoniae* KP5 with the whole genomes of other representative NDM-producing *K. pneumoniae* strains available in the GenBank database (Table S1).

### Nucleotide sequence accession numbers

The whole-genome sequences of these strains were deposited with the National Center for Biotechnology Information (NCBI) under the Bio-Project PRJNA813961. The two plasmids sequences were submitted to the GenBank database and can be found under accession numbers OQ785270-OQ785271.

## Results

### Characteristics of bacterial isolates

Among the isolates, 21 CRKP strains were continuously isolated from June to December in a local tertiary care hospital; 10 were from sputum samples, 4 from urine samples, 3 from wound swabs, 2 from abscess samples and 1 each from a rectal swab and a bronchial washing fluid. Antimicrobial susceptibility testing showed that the 21 CRKP isolates were resistant to ampicillin, carbapenems, cephems, chloramphenicol, fluoroquinolone, macrolides and trimethoprim/sulfamethoxazole, whereas all isolates were susceptible to amikacin, colistin, gentamicin and tetracycline, except for the KR20-0408 isolate, which was resistant to tetracycline (Table 1).

**Table 1:** Antimicrobial susceptibility profiles of 21 NDM-1-producing *K. pneumoniae* isolates in this study

Isolates	MIC, mg/L																
	AMI	AMP	FOX	CAZ	CTX	IMI	MER	ETP	DOR	NAL	CIP	SXT	CHL	TET	GEN	COL	AZI
KR20-0358	< 4	> 64	> 32	> 16	> 32	> 8	16	32	8	> 128	> 16	> 16/304	> 32	4	< 1	< 2	> 32
KR20-0408	< 4	> 64	> 32	> 16	> 32	> 8	8	32	8	> 128	> 16	> 16/304	> 32	64	< 1	< 2	> 32
KR20-0410	< 4	> 64	> 32	> 16	> 32	> 8	8	32	8	> 128	> 16	> 16/304	> 32	4	< 1	< 2	> 32
KR20-0389	< 4	> 64	> 32	> 16	> 32	> 8	8	16	8	> 128	> 16	> 16/304	> 32	4	< 1	< 2	> 32
KR20-0405	8	> 64	> 32	> 16	> 32	> 8	> 32	> 32	> 32	> 128	> 16	> 16/304	> 32	8	< 1	< 2	32
KR20-0406	< 4	> 64	> 32	> 16	> 32	> 8	16	32	8	> 128	> 16	> 16/304	> 32	4	< 1	< 2	32
KR20-0411	< 4	> 64	> 32	> 16	> 32	> 8	16	32	16	> 128	> 16	> 16/304	> 32	4	< 1	< 2	> 32
KR20-0412	< 4	> 64	> 32	> 16	> 32	> 8	16	32	16	> 128	> 16	> 16/304	> 32	4	< 1	< 2	32
KR20-0413	< 4	> 64	> 32	> 16	> 32	> 8	16	32	8	> 128	> 16	> 16/304	> 32	4	< 1	< 2	> 32
KR20-0357	< 4	> 64	> 32	> 16	> 32	> 8	8	32	8	> 128	> 16	> 16/304	> 32	4	< 1	< 2	> 32
KR20-0381	< 4	> 64	> 32	> 16	> 32	> 8	> 32	> 32	> 32	> 128	> 16	> 16/304	> 32	4	< 1	< 2	> 32
KR20-0415	< 4	> 64	> 32	> 16	> 32	> 8	8	32	8	> 128	> 16	> 16/304	> 32	4	< 1	< 2	> 32
KR20-0421	< 4	> 64	> 32	> 16	> 32	> 8	8	32	8	> 128	> 16	> 16/304	> 32	4	< 1	< 2	> 32
KR20-0416	< 4	> 64	> 32	> 16	> 32	> 8	16	32	8	> 128	> 16	> 16/304	> 32	4	< 1	< 2	32
KR20-0423	< 4	> 64	> 32	> 16	> 32	> 8	8	32	8	> 128	> 16	> 16/304	> 32	4	< 1	< 2	> 32
KR20-0426	8	> 64	> 32	> 16	> 32	> 8	8	32	8	> 128	> 16	> 16/304	> 32	4	< 1	< 2	> 32
KR20-0427	< 4	> 64	> 32	> 16	> 32	> 8	> 32	> 32	> 32	> 128	> 16	> 16/304	> 32	8	< 1	< 2	> 32
KR20-0428	< 4	> 64	> 32	> 16	> 32	> 8	> 32	> 32	> 32	> 128	> 16	> 16/304	> 32	4	< 1	< 2	32
KR20-0430	< 4	> 64	> 32	> 16	> 32	> 8	> 32	> 32	> 32	> 128	> 16	> 16/304	> 32	8	< 1	< 2	> 32
KR20-0434	< 4	> 64	> 32	> 16	> 32	> 8	> 32	> 32	> 32	> 128	> 16	> 16/304	> 32	4	< 1	< 2	32
KR20-0369	< 4	> 64	> 32	> 16	> 32	> 8	> 32	> 32	> 32	> 128	> 16	> 16/304	> 32	4	< 1	< 2	32

### PFGE analysis

**Fig. 1:**
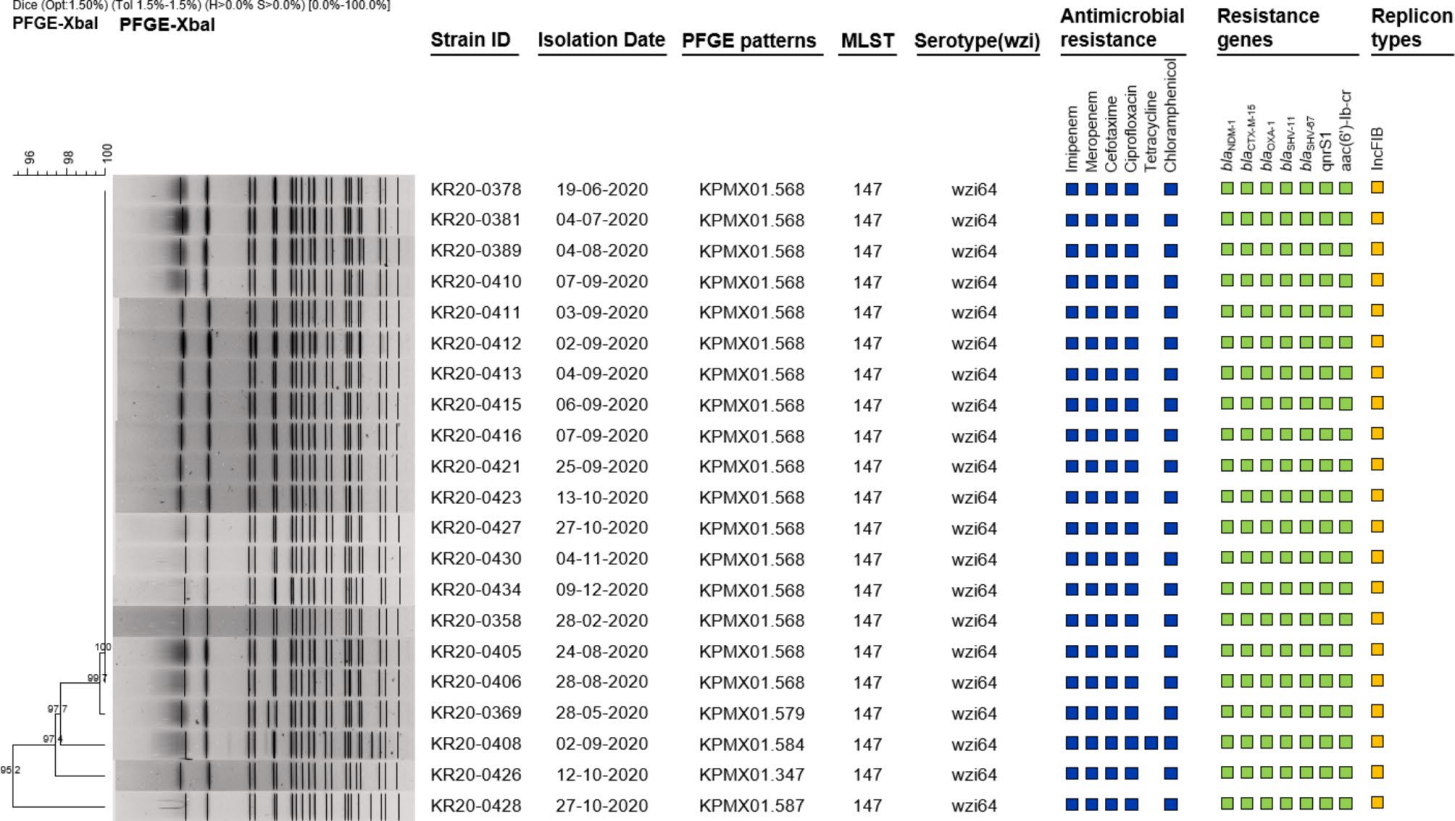
**Molecular epidemiology of** ***Klebsiella pneumoniae*** **ST147-KL64 isolates possessing** ***bla***_**NDM−1**_ **in Korea in 2020** This dendrogram was constructed with BioNumerics (Applied-Maths, Belgium) by utilizing the unweighted-pair group method with arithmetic means and a Dice coefficient (1.5% optimization and 1.5% position tolerance). Strain ID, isolation date, PFGE patterns, MLST, *wzi* type, antibiotic susceptibility, antimicrobial resistance genes and plasmid replicon types are indicated

All isolates were divided into five different PFGE patterns and were closely related at 95.2% of the minimum similarity value (Fig. 1). Seventeen isolates were clustered together into the KPMX01.568 pattern. Four isolates were divided into four different patterns (KPMX01.347, KPMX01.579, KPMX01.584, and KPMX01.587), differing by only one band.

### Genomic analysis

According to the WGS data and MLST scheme of *K. pneumoniae*, all 21 NDM-1-producing CRKP isolates were identified as ST147 and confirmed to have the same capsular KL64 serotype. Additionally, virulence genes were confirmed to be involved in yersiniabactin (irp1, irp2, ybtAEPQSTUX, and fyuA) and fimbrial adhesion (mrkABCDFHIJ). All 21 ST147-KL64 CRKP isolates coharbored additional resistance determinants, including beta-lactam (*bla*_OXA−1_, *bla*_CTX−M−15_, *bla*_SHV−11_, and *bla*_SHV−67_), quinolone (*aac(6’)-Ib-cr*, *qnrS1*, *OqxA*, and *OqxB*), chloramphenicol (*catB3*), sulfamethoxazole (*sul1*), and trimethoprim (*dfrA12*) resistance genes, as well as mutations in the quinolone resistance-determining regions (QRDRs) of *gyrA* (S83I) and *parC* (S80I) (Fig. 1).

### Plasmid analysis

**Fig. 2:**
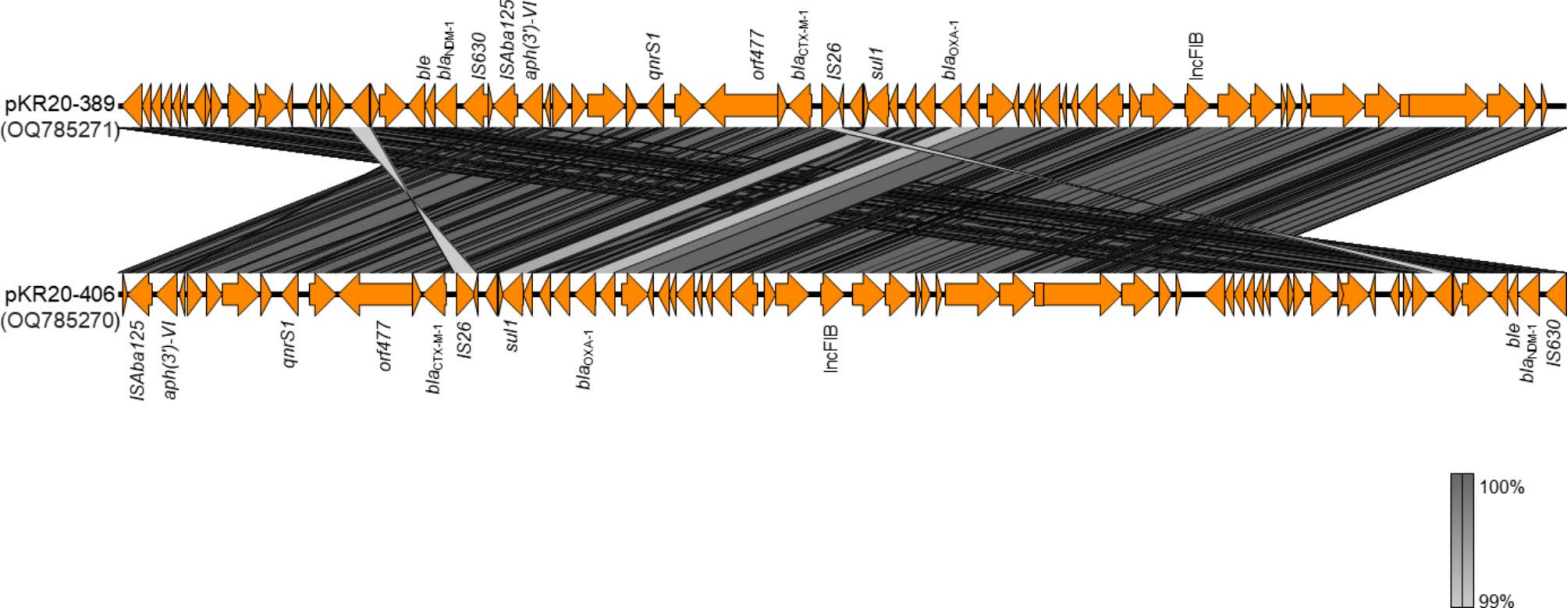
**Sequence alignment analysis of IncFIB** ***bla***_**NDM−1**_ **plasmids** Linear alignment of the sequences of two representative *bla*_NDM−1_ harboring *K. pneumoniae* strains in this study. The gray shaded area indicates the nucleotide similarity between the corresponding genetic loci in each plasmid

According to whole-genome sequence analysis, 21 ST147-KL64 CRKP isolates shared a common genetic structure. The plasmid incompatibility group of the 21 NDM-1-producing ST147 *K. pneumoniae* included an IncFIB replicon plasmid approximately 54 kb in size (Fig. 2). The representative ST147-KL64 CRKP genome showed that the flanking regions of the *bla*_NDM−1_ gene surrounded the insertion sequences *ISAba125* and *IS630* upstream and downstream of the bleomycin resistance gene (*ISAba125*-*IS630*-*bla*_NDM−1_-*ble*_MBL_). The other resistance region was carried by *IS26* and *orf477* on the transposon unit *orf477*-*bla*_CTX−M−15_-*IS26* (Fig. 2). This plasmid carried other beta-lactamase genes and those conferring resistance to quinolone, chloramphenicol, and sulfamethoxazole. Attempts to transfer these NDM-1-harboring plasmids by conjugation were unsuccessful. However, the long- and short-read sequencing results confirmed that the *bla*_NDM−1_ gene was located on the IncFIB replicon plasmid.

### Clonal relatedness of bacterial isolates

**Fig. 3:**
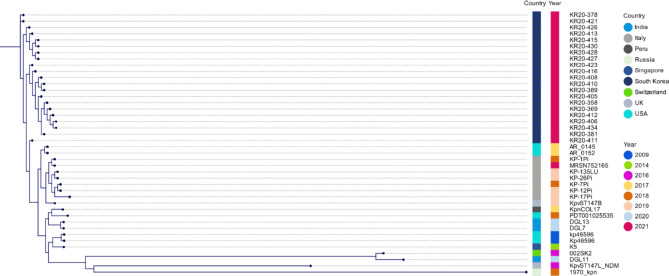
**Phylogenetic tree compared to NDM-1-producing** ***K. pneumoniae*** **ST147-KL64 strains** The phylogenetic tree was constructed with CSI phylogeny 1.4 (https://cge.cbs.dtu.dk/services/CSIPhylogeny/) using the genome of *K. pneumoniae* KP5 as a reference

The phylogenetic analysis of SNPs demonstrated that all strains, including 20 reference strains, differed from each other by 2-2247 SNPs. All *K. pneumoniae* isolates carrying *bla*_NDM_ were typed as ST147. Notably, 21 ST147-KL64 CRKP isolates from this study were shown to be significant phylogroups with limited SNP (2–27) divergences (Fig. 3). Whole-genome comparison showed that 7 reference strains obtained from clinical samples in Italy and the USA (KP-12Pi, KP-26Pi, KP-135LU, KP-1Pi, AR_0145, AR_0152 and MRSN752165) are closely related, differing by 68–109 SNPs.

## Discussion

From 2017 to 2020, the Korean Antimicrobial Resistance Monitoring System for CRE infections reported that *K. pneumoniae* was the most frequent pathogen (64.3%), followed by *Escherichia coli* (18%) and *Enterobacter cloacae* (3.8%); among these pathogens, *bla*_KPC_ (75.4%) and *bla*_NDM_ (18%) were the predominant carbapenemase genotypes [2].

The first identification of nosocomial infections associated with NDM-1 producing *K. pneumoniae* were reported in the Republic of Korea in 2010 [14]. *K. pneumoniae* was known as one of the causes of nosocomial infections, and it transferred rapidly due to the efficiency of colonization and acquired resistance to antibiotics [15]. In this study, we demonstrate that *K. pneumoniae* ST147-KL64 isolates harboring *bla*_NDM−1_ have spread in a local tertiary care hospital in the Republic of Korea.

Attempts to transfer these NDM-1-harboring IncFIB plasmids by conjugation were unsuccessful, in agreement with the absence of four conjugation modules (oriT, relaxase, type IV coupling protein [T4CP], and T4SS) from their backbone. However, the results showed that both PFGE and SNP analyses results identified genetically highly related isolates (> 95.2% similarity and 2–27 SNPs differences). Therefore, we confirmed that this nosocomial infection was related to the clonal spread of NDM-1 producing *K. pneumoniae* rather than the horizontal transmission of plasmids.

The draft genomes revealed a shared plasmid backbone (> 99% nucleotide identity) among the NDM-1-producing ST147-KL64 isolates from these nosocomial infections (Fig. 2). The *bla*_NDM−1_ gene was encoded on an IncFIB plasmid, although *bla*_NDM−1_ was bracketed by two insertion sequences, *ISAba125* and *IS630*, which belong to the *IS30* and *IS630* families, respectively.

IncFIB plasmids harboring *bla*_NDM−1_ were highly homologous with pAR_0145, 7008.20-NDM1 and pM321-NDM1 from *K. pneumoniae* isolated from the USA, Switzerland and Myanmar (99% nucleotide identity, GenBank accession numbers CP021941, CP082992 and AP018834) [16, 17]. These plasmids shared a common plasmid backbone with our isolates and possessed a multidrug resistance region harboring *bla*_NDM−1_.

Incorporating public *bla*_NDM_-producing *K. pneumoniae* ST147 data into the analysis revealed that these isolates formed a single cluster, suggesting the acquisition of *bla*_NDM_ by a clonally spreading *K. pneumoniae* ST147-KL64 strain in one hospital. In addition, these isolates have an identical capsular type, plasmid replicon type, resistance determinant set and mobile genetic element set, which may have been introduced from common sources, implying nosocomial transmission. However, the limited acquisition of NDM-1-producing *K. pneumoniae* ST147-KL64 could not be explained by the spread from humans to humans or contamination of the environment.

An NDM-producing ST147-KL64 clone has been reported annually in other countries, such as the UK, the USA and Italy [18–20]. Recently, several cases have demonstrated the prevalence of NDM-producing *K. pneumoniae* associated with nosocomial infections, including cases in the USA, Pakistan and the Netherlands [9, 20, 21]. A previous study showed that the *K. pneumoniae* ST147 clone has a global distribution and that it has been responsible for several nosocomial outbreaks worldwide [5].

The emergence of the NDM-1-producing *K. pneumoniae* ST147-KL64 clone in a local tertiary care hospital is a concern not only for transmission in hospitals but also for the spread of community-acquired transmission. Thus, increased monitoring is necessary to prevent the dissemination of high-risk clones, and adequate infection control measures against the spread of national and transnational infections, especially those targeting underlying mechanisms, are needed.

## Conclusions

The results of this study indicated that persistent infections caused by NDM-1-producing *K. pneumoniae* ST147 occurred in a local tertiary care hospital in the Republic of Korea. Our findings indicate that NDM-1-producing *K. pneumoniae* ST147 may have been introduced via a common source, implying nosocomial transmission. Therefore, adequate infection control measures are necessary to prevent the further dissemination by nosocomial and endemic transmission.

### Electronic supplementary material

Below is the link to the electronic supplementary material.

Supplementary data: Table S1. List of the GenBank-archived K. pneumoniae ST147 strains under analysis and Bacterial stains used in this study.

## Data Availability

Not applicable.

## List of abbreviations

CRE: Carbapenem-resistant *Enterobacterales*
NDM: New Delhi metallo-β-lactamase
CRKP: Carbapenem-resistant *Klebsiella pneumoniae*
PFGE: Pulsed-field gel electrophoresis
WGS: Whole-genome sequencing
