# Supplementary material for: Dissemination of the high-risk cloneST147 carbapenem-resistant klebsiella pneumoniae from a local tertiary care hospital in the Republic of Korea
Source: Ann Clin Microbiol Antimicrob. 2023 Aug 24;22:76. doi: 10.1186/s12941-023-00601-2 (PMC10464262; doi:10.1186/s12941-023-00601-2)
Supplement: Supplementary file 1 — Supplementary data: Table S1. List of the GenBank-archived K. pneumoniae ST147 strains under analysis and Bacterial stains used in this study. [file 12941_2023_601_MOESM1_ESM.doc]

**Supplementary data**

**Table S1**. List of the GenBank-archived *K. pneumoniae* ST147 strains under analysis and Bacterial stains used in this study.

| Strain | Genotype | GenBank accession number | Reference |
| --- | --- | --- | --- |
|  | | |  |
| AR_0145 | ST147, wzi64, *bla*_NDM-1_, *bla*_CTX-M-15_ | CP021939 | Unpublished |
| AR_0152 | ST147, wzi64, *bla*_NDM-1_, *bla*_CTX-M-15_ | CP021944 | Unpublished |
| KP-1Pi | ST147, wzi64, *bla*_NDM-1_, *bla*_CTX-M-15_ | CP071027 | Unpublished |
| MRSN752165 | ST147, wzi64, *bla*_NDM-1_, *bla*_CTX-M-15_ | CP074087 | Unpublished |
| KP-135LU | ST147, wzi64, *bla*_NDM-1_, *bla*_CTX-M-15_ | CP070890 | Di Pilato,V., et al |
| KP-26Pi | ST147, wzi64, *bla*_NDM-1_, *bla*_CTX-M-15_ | CP072925 | Di Pilato,V., et al |
| KP-7Pi | ST147, wzi64, *bla*_NDM-1_, *bla*_CTX-M-15_ | JAFHLG000000000 | Di Pilato,V., et al |
| KP-12Pi | ST147, wzi64, *bla*_NDM-1_, *bla*_CTX-M-15_ | CP072917 | Unpublished |
| KP-17Pi | ST147, wzi64, *bla*_NDM-1_, *bla*_CTX-M-15_ | JAFHLI000000000 | Di Pilato,V., et al |
| KpvST147B | ST147, wzi64, *bla*_NDM-1_, *bla*_CTX-M-15_ | CP040724 | Unpublished |
| KpnCOL17 | ST147, wzi64, *bla*_NDM-1_, *bla*_CTX-M-15_ | CP072905 | Souvorov,A., et al |
| PDT001025535 | ST147, wzi64, , *bla*_NDM-1_ | DAFYSJ000000000 | Unpublished |
| DGL13 | ST147, wzi64, *bla*_NDM-5_, *bla*_CTX-M-15_ | JAJBAB000000000 | Unpublished |
| DGL7 | ST147, wzi64, *bla*_NDM-5_, *bla*_CTX-M-15_ | JAJBAH000000000 | Unpublished |
| Kp46596 | ST147, wzi64, *bla*_NDM-1_, *bla*_CTX-M-15_ | CP059312 | Unpublished |
| K5 | ST147, wzi116, *bla*_NDM-1_, *bla*_CTX-M-15_ | JAHLXX000000000 | Unpublished |
| 002SK2 | ST147, wzi420, *bla*_NDM-9_, *bla*_CTX-M-15_ | CP025515 | Unpublished |
| DGL11 | ST147, wzi420, *bla*_NDM-5_, *bla*_CTX-M-15_ | JAJBAD000000000 | Unpublished |
| KpvST147L_NDM | ST147, wzi23, *bla*_NDM-1_, *bla*_CTX-M-15_ | MZMY00000000 | Unpublished |
